# DREAMER: Rapid and Simultaneous Multiple Contrast Magnetic Resonance Imaging of Solid and Soft Tissue

**DOI:** 10.1002/mrm.70140

**Published:** 2025-10-29

**Authors:** Brian‐Tinh Duc Vu, Nada Kamona, Felix W. Wehrli, Rajiv S. Deshpande, Hee Kwon Song, Allison Hu, Emily Baccaglini, Scott P. Bartlett, Chamith S. Rajapakse

**Affiliations:** ^1^ Department of Radiology, Perelman School of Medicine University of Pennsylvania Philadelphia Pennsylvania USA; ^2^ Department of Bioengineering, School of Engineering and Applied Sciences University of Pennsylvania Philadelphia Pennsylvania USA; ^3^ Division of Plastic, Reconstructive and Oral Surgery Children's Hospital of Philadelphia Philadelphia Pennsylvania USA; ^4^ Department of Orthopaedic Surgery, Perelman School of Medicine University of Pennsylvania Philadelphia Pennsylvania USA

**Keywords:** multi‐contrast MRI, solid‐state imaging, craniofacial imaging, pulse sequence, pediatric imaging

## Abstract

**Purpose:**

Pediatric craniofacial imaging may involve examination of both the skull and brain tissues via CT and MRI, respectively. DREAMER (Dual Repetition and Echo Acquisition with Multi‐contrast Encoding and Reconstruction) simultaneously acquires solid‐ and soft‐tissue images, potentially providing a rapid, high‐resolution, and radiation‐free protocol whenever bone‐selective, T_1_w, and T_2_w images are required.

**Methods:**

The DREAMER sequence combines a solid‐state MRI method with phase‐based T_2_ encoding to produce a multi‐contrast signal model that enables retrospective customization of image contrast weighting. DREAMER is paired with an iterative image reconstruction algorithm for accelerated and high‐resolution structural imaging of solid‐ and soft‐tissue compartments. Two healthy adult volunteers and two pediatric patients were scanned at 3 T to qualitatively compare soft‐tissue DREAMER image contrasts with their corresponding clinical standards, T_1_w MPRAGE and T_2_w fast spin‐echo (FSE). Two patients also underwent clinical CT to compare the bone‐selective images and skull renderings.

**Results:**

DREAMER images are self‐registered, high‐resolution, and spatially isotropic. The bone‐selective, T_1_w, and T_2_w images approximate the image contrasts and structural imaging capabilities of their corresponding clinical standards (CT, T_1_w MPRAGE, and T_2_w FSE). Unlike the standard techniques, DREAMER imaging occurs at a single scanner using a single pulse sequence.

**Conclusion:**

DREAMER combines mechanisms for solid‐ and multiple‐contrast soft‐tissue imaging into a single scan. For craniofacial imaging, DREAMER may consolidate CT and MRI demand, reduce exposure to ionizing radiation, decrease patient examination and wait times, and simplify the radiological workflow.

## 1 Introduction

CT and MRI are diagnostic mainstays of modern radiology with complementary properties [1, 2]. In CT, highly attenuating solid tissues, like densely calcified bone, are depicted with high signal intensity and excellent contrast [3]. Conventional MRI examinations, typically comprised of several pulse sequences jointly aiming to determine the underlying pathology [4], may obtain a variety of image contrasts relating to direct measurement or weighting of the physical properties of soft tissue [5, 6].

CT and MRI have different limitations. For example, CT scans are rapid (∼30 s), but MRI examinations are comparatively slow because they are comprised of multiple pulse sequences [7]. Unlike MRI, CT uses ionizing radiation, which is known to increase the risk of cancer in children, who are more sensitive to ionizing radiation than adults [8].

Certain clinical presentations involving craniofacial malformations in pediatric patients may benefit from ordering both CT and MRI. For example, syndromic craniosynostoses may involve the fusion of multiple skull sutures and underlying cerebral anomalies [9, 10]. In fibrous dysplasia, fibrous connective tissue replaces healthy bone tissue and may compress neurological structures [11, 12]. Finally, for pediatric craniofacial trauma, CT and MRI may be used in conjunction to assess both skull and parenchymal injuries, respectively [13, 14]. Ideally, these cases would use CT to assess the bony anatomy and T_1_w and T_2_w MRI to produce exquisite soft tissue contrast and detect gross abnormalities. Imaging with both modalities may present economical and logistical challenges in practice [15, 16], and hence, a one‐stop‐shop protocol may be desirable.

The first step towards constructing an improved and consolidated protocol for pediatric craniofacial imaging is to combine fundamental and desirable properties of CT (bone‐selective, rapid, high‐resolution) and MRI (enhanced and multiple soft‐tissue contrast, no ionizing radiation) in a single 3D scan without the requirement of nonstandard clinical hardware. Such a method would produce an efficient mode of scanning that obviates the use of ionizing radiation, simplifies the clinical workflow, and consolidates demand for high‐resolution clinical imaging, potentially avoiding the economic and logistical burden of imaging with two different scanners [15, 16].

Existing techniques fall short of satisfying these requirements. Phase‐contrast and photon‐counting CT may enhance soft tissue contrast relative to conventional CT. However, they require nonstandard hardware, have so far seen limited clinical use, and use ionizing radiation [17, 18]. “Black‐bone MRI” techniques use gradient echo (GRE) sequences with small RF flip angles to acquire 3D soft‐tissue images. The resulting proton‐density weighted (PDw) images have relatively uniform soft tissue signal, but signal from bone water, water hydrogen‐bonded to the collagen matrix, and the major source of detectable protons in bone [19], decays before it can be measured. This is because bone water T_2_ (∼300–400 μs) is several times shorter than the TE used for signal measurement [20]. For black‐bone techniques, bone identification is based on the absence of signal, which conflates bone with air [21, 22]. Furthermore, PDw images are not as commonly used as T_1_‐weighted or T_2_‐weighted (T_1_w and T_2_w) images, which would have to be acquired in succession, prolonging examinations. Solid‐state MRI techniques, like ultrashort echo time [23, 24] (UTE) and zero‐echo time [25, 26] (ZTE) sequences, directly capture the signal from short‐T_2_ protons of osseous tissues. However, the resulting soft‐tissue images are typically limited to either PDw or T_1_w contrast. Finally, data‐driven synthetic methods may generate CT from MRI [27, 28, 29, 30] but the training requires large datasets to cover the wide variability of patient phenotypes, demographics, and pathologies. Even when large datasets are available, issues regarding generalizability and out‐of‐distribution samples may occur [31, 32]. Hence, medical image synthesis is costly and challenging.

As a first step towards creating a craniofacial imaging protocol that combines several of the beneficial properties of CT and MRI, the present study proposes a new MRI pulse sequence (DREAMER: Dual Repetition and Echo Acquisition with Multi‐contrast Encoding and Reconstruction) to perform simultaneous solid (bone‐selective) and multi‐contrast soft tissue (T_1_w and T_2_w) imaging in a single scan. The proposed DREAMER pulse sequence uses conventional MRI hardware and may obviate the need for separate CT and MRI exams. Because the multi‐contrast encoding is based on the T_2_ relaxation properties of tissues and is not synthetic or generative, it obviates the accumulation of large training datasets and confrontation with out‐of‐distribution data. DREAMER leverages the availability of multi‐channel receiver coils [33] to substantially reduce the scan duration [34]. It is compatible with model‐based image reconstruction techniques [35, 36], enabling fast and concurrent solid‐ and soft‐tissue imaging. The DREAMER outputs (i.e., 3D bone‐selective, T_1_w, and T_2_w images) are self‐registered, high‐resolution (1 mm^3^), and spatially isotropic, potentially producing a one‐stop shop for structural solid and soft tissue craniofacial imaging. Parts of this work were previously presented in abstract form at the ISMRM Annual Meeting and Exhibition, Honolulu, Hawaii, 2025 [37, 38].

## 2 Methods

### 2.1 Partially RF‐Spoiled Gradient Echo: Concurrent T_1_w and T_2_w Imaging

Because of their short TRs, GRE sequences always achieve some T_1_‐weighting, provided that the RF flip angle is reasonably large. Whether an additional T_2_‐weighting is achieved depends on the prescribed RF phase schedule ϕ(n), which dictates how the residual magnetization at the end of each TR contributes to the signal in subsequent TR cycles (n denotes the RF pulse index). For the magnetization to achieve steady state, a single RF phase increment θ, with −180°≤θ<180°, may be used to determine the phase schedule by the recurrence relation:

(1) ϕ(n)=ϕ(n−1)+nθ

For example, an RF‐spoiled GRE, using an RF phase increment of 117°, achieves pure T_1_‐weighting because the selection of θ causes the residual magnetization from previous TRs to accumulate incoherently [39]. An unbalanced SSFP sequence (θ=0° or, more generally, a phase schedule of ϕ(n)=nθ) uses a train of RF pulses that is phase coherent and achieves an image weighting that is a function of both T_1_ and T_2_ [40].

**FIGURE 1:**
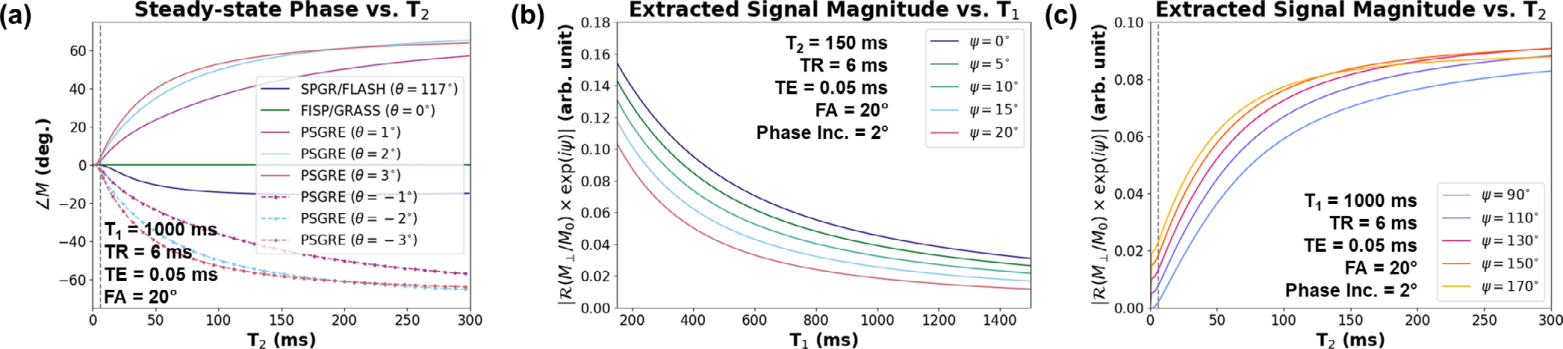
Bloch simulations of a partially spoiled gradient echo sequence (PSGRE). (a) Steady‐state signal phase versus T_2_ for various RF phase increments. Commonly used RF‐spoiled gradient echo (“SPGR” or “FLASH”; θ=117°) and steady‐state free precession (“FISP” or “GRASS”; θ=0°) sequences are plotted for reference. The vertical gray dotted line indicates the simulated TR. For tissues with T_2_ ≫ TR, a small, nonzero RF phase increment produces a signal phase which is a monotonic function of tissue T_2_. The signal phases of conventional GRE sequences (SPGR/FLASH and FISP/GRASS) do not encode T_2_. Flipping the sign of the small RF phase increment flips the sign of the steady‐state signal phase. (b) T_1_w images (i.e., signal magnitude decreasing with increasing tissue T_1_) are encoded in the signal for a small interval of phase modulation values (∼0°–20°). This T_1_w signal is extracted by projecting the complex‐valued signal onto the real axis after a phase modulation ψ. (c) Likewise, T_2_w images (i.e., signal magnitude increasing with increasing tissue T_2_) are encoded for a separate interval of phase modulation values (∼90°–170°).

A partially RF‐spoiled gradient echo (PSGRE) introduces a small amount of phase incoherence by reducing the RF phase increment to a small, nonzero value (e.g., 1°–4°). In this small RF phase increment regime, tissues with longer T_2_ accumulate a larger phase (i.e., the measured signal phase monotonically increases with increasing tissue T_2_), thereby encoding an additional T_2_‐weighting along the phase of the signal [41] (Figure 1a). Bloch simulations were performed using Sycomore [42]. The measured steady‐state signal, which consists of a superposition of FID, spin‐echo, and stimulated‐echo magnetization components, is denoted as the *multi‐contrast signal* or *multi‐contrast image* because it stores both T_1_w and T_2_w magnetization components in the signal along different values of the signal phase. This phenomenon forms the basis for simultaneous T_1_w and T_2_w imaging [43].

Extending the work of Tamada et al. [43], the selection of a specific image contrast is performed by rotation of the complex‐valued multi‐contrast image x by a phase modulation angle ψ before projection on the real axis:

(2) $$\begin{array}{c} x_{\psi }=\left|R\left(xe^{i\psi }\right)\right| \end{array}$$

The operation in Equation (2) is called *contrast extraction*. $x_{\psi }$ denotes the extracted contrast, which was encoded along an angle ψ measured from the real axis. R(u) denotes the voxel‐wise real value of an arbitrary complex‐valued image u, and |u| is the voxel‐wise absolute value of u.

Figure 1b,c illustrates how contrast extraction can achieve T_1_‐ and T_2_‐weighting. For the simulated PSGRE parameters, T_1_‐weighting (signal magnitude decreasing with increasing T_1_) is attained for a small interval of phase modulation values (0°≤ψ≤20°). Likewise, T_2_‐weighting (signal magnitude increasing with increasing T_2_) is achieved on a separate interval (90°≤ψ≤170°). Because the phase modulation ψ is tunable at the point of reconstruction, T_1_‐ and T_2_‐weighting may be retrospectively adjusted to suit the needs of the user. Notably, all unique image contrasts are extracted for phase modulation values of 0°≤ψ<180°. This is demonstrated by showing that $x_{\psi }$ is *π*‐periodic:

(3) $$\begin{array}{cc} x_{\psi +\pi } & =\left|R\left(xe^{i\left(\psi +\pi \right)}\right)\right|=\left|R\left(-xe^{i\psi }\right)\right|\\ & =\left|-1\cdot R\left(xe^{i\psi }\right)\right|=\left|R\left(xe^{i\psi }\right)\right|=x_{\psi } \end{array}$$

Thus, by restricting phase modulation values to 0°≤ψ<180°, all soft tissue contrasts that may be reconstructed from the phase‐based encoding technique of a PSGRE will be included.

In practice, the steady‐state magnetization is corrupted by a spatially varying background phase shift, caused by inhomogeneities in the static field and RF excitation. The background phase is modeled by a pointwise multiplicative term $e^{\mathrm{i\varphi}\left(r\right)}$, with φ=φ(r) serving as a gentle reminder that the background phase may assume a different value at each voxel [41]. The forward model for a phase‐corrupted PSGRE acquisition is

(4) $$\begin{array}{c} y=\mathrm{FS}e^{\mathrm{i\varphi}\left(r\right)}x \end{array}$$

S denotes pointwise multiplication by coil sensitivities, F encodes the Fourier sampling trajectory of the PSGRE sequence, and y is the acquired multi‐coil k‐space dataset. A naïve reconstruction using coil sensitivities S would yield not x, but $e^{\mathrm{i\varphi}\left(r\right)}x$, a phase‐corrupted version of the multi‐contrast image. Without knowledge of the background phase, $e^{\mathrm{i\varphi}\left(r\right)}$ cannot be separated from x. Because the image contrasts are stored in the phase of x, a spatially varying background phase disrupts the contrast extraction method described by Equation (2).

To resolve the issue of the background phase, a two‐pass strategy is implemented in which the second (negative) pass flips the sign of the RF phase increment used in the first (positive) pass [41, 43]. Figure 1a shows that flipping the sign of θ also flips the sign of the steady‐state signal phase. As a result, the signal phase of the negative pass monotonically decreases for increasing T_2_, and the underlying multi‐contrast image, uncorrupted by the background phase, is $x^{*}$, which denotes voxel‐wise complex conjugation of the multi‐contrast image x. The corresponding forward models for positive and negative passes are.

(5a) $$\begin{array}{c} y_{+}=\mathrm{FS}x_{+}=\mathrm{FS}e^{\mathrm{i\varphi}\left(r\right)}x \end{array}$$

(5b) $$\begin{array}{c} y_{-}=\mathrm{FS}x_{-}=\mathrm{FS}e^{\mathrm{i\varphi}\left(r\right)}x^{*} \end{array}$$

Subscripts denote association with the positive and negative passes. $x_{+}=e^{\mathrm{i\varphi}\left(r\right)}x$ and $x_{-}=e^{\mathrm{i\varphi}\left(r\right)}x^{*}$ are phase‐corrupted multi‐contrast images from the positive and negative passes, respectively. One crucial assumption of this model is that the background phase is identical in both acquisitions. The phase‐corrupted positive and negative pass images are computed using model‐based image reconstruction:

(6a) $$\begin{array}{c} x_{+}=\underset{u}{\text{argmin}}{\left\|\mathrm{FSu}-y_{+}\right\|}_{2}^{2} \end{array}$$

(6b) $$\begin{array}{c} x_{-}=\underset{v}{\text{argmin}}{\left\|\mathrm{FSv}-y_{-}\right\|}_{2}^{2} \end{array}$$

Model‐based image reconstruction with coil sensitivities enables undersampled [36] and arbitrary sampling schemes [34] with the PSGRE signal model. The background phase is explicitly calculated from the phases of $x_{+}$ and $x_{-}$,

(7) $$\begin{array}{c} e^{\mathrm{i\varphi}\left(r\right)}={\left(\frac{x_{+}}{x_{-}^{*}}\right)}^{\frac{1}{2}} \end{array}$$

and the multi‐contrast image is retrieved by averaging the phase‐corrected images from the positive and negative passes,

(8) $$\begin{array}{c} x=\frac{1}{2}\left[x_{+}e^{-\mathrm{i\varphi}\left(r\right)}+{\left(x_{-}e^{-\mathrm{i\varphi}\left(r\right)}\right)}^{*}\right] \end{array}$$

Contrast extraction by Equation (2) can then proceed without hindrance from the background phase. Figure 3a illustrates the steps of converting positive and negative pass images to extracted T_1_w and T_2_w contrasts.

### 2.2 Dual‐Echo UTE: Bone‐Selective Imaging

**FIGURE 2:**
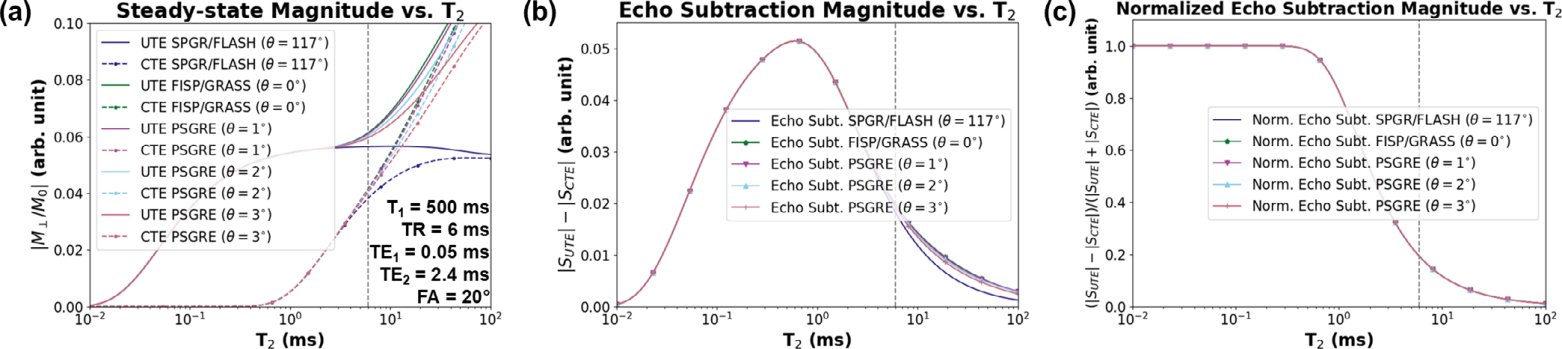
Bloch simulations of dual‐echo UTE sequences for various RF phase increments. Note that T_2_ is plotted on a logarithmic scale and the vertical dotted gray line indicates the simulated TR. The application of RF pulses is assumed to be instantaneous (i.e., no loss in excitation efficiency when applied to short‐T_2_ species). (a) For tissues with short T_2_ (T_2_ ≪ TR; e.g., bone), the steady‐state signal magnitude is independent of the RF phase increment. Soft tissues with long T_2_ (T_2_ ≫ TR; e.g., white/gray matter, cerebrospinal fluid, fat) accumulate phase based on their T_2_ value and thus exhibit a signal magnitude dependency on the RF phase increment. (b) An echo subtraction isolates short‐T_2_ signals but may leave some long‐T_2_ signals unsuppressed. (c) A normalized echo subtraction further enhances short‐T_2_ signals while suppressing long‐T_2_ signals. Furthermore, a normalized echo subtraction exhibits no signal dependency on the RF phase increment.

While the PSGRE signal mechanism encodes a T_2_‐weighting along the signal phase of long‐T_2_ species, this mechanism fails for short‐T_2_ species like collagen‐bound bone water. In a GRE, short‐T_2_ magnetization (T_2_ ≪ TR) is briefly coherent immediately following an RF pulse and experiences a rapid and near‐complete decay before the pulse of the subsequent TR [23, 24, 44]. Because little or no residual magnetization remains by the end of the TR, the steady‐state signal behavior of short‐T_2_ magnetization is independent of the prescribed RF phase schedule of any GRE sequence. This is shown by simulation data (Figure 2a), which assumes ideal RF excitation efficiency (i.e., instantaneous application of an RF pulse) [45, 46]. In the simulation, the signal magnitude of long‐T_2_ species (T_2_ ≫ TR) exhibits a dependence on the RF phase increment, while that of short‐T_2_ species does not. Instead, the short‐T_2_ signal magnitude is predominantly a function of the transverse decay occurring within a single TR and the TE used to sample the rapidly decaying signal.

A dual‐echo UTE sequence is designed to explicitly probe the intra‐TR decay of short‐T_2_ signal. Like a GRE, this sequence also uses a regularly spaced train of RF pulses, but two data acquisitions are performed per TR: one at an ultrashort echo time (UTE; $x_{\mathrm{UTE}}$) and another at a conventional echo time (CTE; $x_{\mathrm{CTE}}$). The UTE acquisition is performed with minimal delay after the RF pulse (∼30–60 μs), while the CTE acquisition uses a delay 4–10× the T_2_ of bone water (which is ∼300–400 μs [20]), but substantially less than the T_2_ of soft tissue. The long‐T_2_ components undergo negligible signal decay and appear with approximately the same signal magnitude in both echoes. Ignoring the effects of proton density on the signal magnitude, an echo subtraction $\left|x_{\mathrm{UTE}}\right|-\left|x_{\mathrm{CTE}}\right|$ suppresses long‐T_2_ signal and highlights short‐T_2_ components (Figure 2b). Notably, it also reduces the dependence of the subtraction signal on the RF phase increment.

Tissue species with both a long‐T_2_ and high proton density, like fat, also appear with high signal intensity in a standard echo subtraction. This is because a large proton density compensates for a small but nonzero decay in transverse magnetization between the ultrashort and conventional TEs. Since bone has low proton density (∼20% by volume [47]), the presence of a long‐T_2_, high proton density species reduces bone specificity [23]. A *normalized* echo subtraction removes proton density weighting [45] and computes a bone‐selective image $x_{s}$:

(9) $$\begin{array}{c} x_{s}=\frac{\left|x_{\mathrm{UTE}}\right|-\left|x_{\mathrm{CTE}}\right|}{\left|x_{\mathrm{UTE}}\right|+\left|x_{\mathrm{CTE}}\right|} \end{array}$$

This operation further enhances short‐T_2_ species and minimizes the dependency of the bone‐selective image signal on the RF phase increment (Figure 2c).

Alternatively, to avoid enhancement of noise from air, which results from the nominal division by zero, a weighted least‐squares method [48] is used to compute the normalized echo subtraction:

(10) $$\begin{array}{c} x_{s}=\underset{u}{\text{argmin}}{\left\|W^{\frac{1}{2}}\left(\left|x_{\mathrm{UTE}}\right|+\left|x_{\mathrm{CTE}}\right|\right)u-W^{\frac{1}{2}}\left(\left|x_{\mathrm{UTE}}\right|-\left|x_{\mathrm{CTE}}\right|\right)\right\|}_{2}^{2} \end{array}$$

W is a set of weights from convolving a low‐pass filter (e.g., a Hanning window) with the standard echo subtraction $\left|x_{\mathrm{UTE}}\right|-\left|x_{\mathrm{CTE}}\right|$. Equation (10) is evaluated by a conjugate gradient method [49]. Unlike the method of Equation (9), the method of Equation (10) uses weights W to localize voxels containing actual signal from tissue. When used in an iterative method like the conjugate gradient, these weights manipulate the convergence behavior [50] of the algorithm to emphasize the emergence of high intensity signal from bone and discourage signal from voxels containing air. Early termination of the conjugate gradient method yields images which are highly specific to bone while retaining the long‐T_2_ signal suppression properties of a normalized echo subtraction [48, 51].

Finally, because $x_{s}$ is derived from both $x_{\mathrm{UTE}}$ and $x_{\mathrm{CTE}}$, it is beneficial to perform joint reconstruction and regularization of the UTE and CTE datasets:

(11) $$\begin{array}{cc} \left(x_{\mathrm{UTE}},x_{\mathrm{CTE}}\right) & =\underset{\left(u,v\right)}{\text{argmin}}{\left\|\mathrm{FSu}-y_{\mathrm{UTE}}\right\|}_{2}^{2}\\ & \;+{\left\|\mathrm{FSv}-y_{\mathrm{CTE}}\right\|}_{2}^{2}+R\left(u,v\right)\; \end{array}$$

**FIGURE 3:**
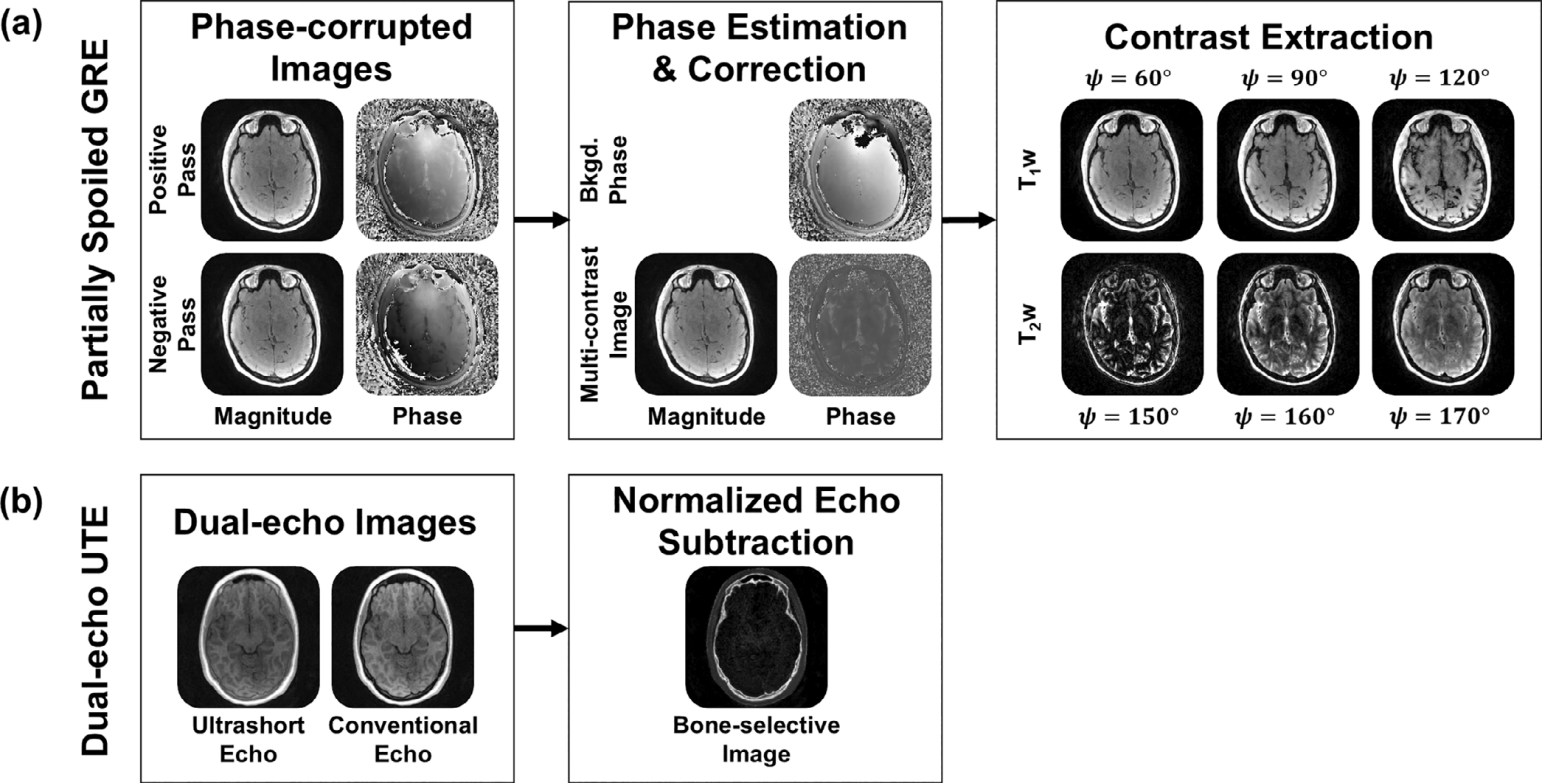
Processing steps of partially spoiled gradient echo (PSGRE) and dual‐echo ultrashort echo (UTE) pulse sequences, the parent sequences of DREAMER. (a) PSGRE sequences acquire multi‐contrast images with tunable T_1_‐ and T_2_‐weighted contrast. Positive and negative passes are used to remove the background phase and recover the multi‐contrast image. The phase modulation parameter ψ, chosen during image reconstruction, dictates contrast extraction of T_1_w and T_2_w soft‐tissue images. (b) Dual‐echo UTE sequences acquire two images with and without bone signal (ultrashort and conventional echo images, respectively). A normalized echo subtraction selectively enhances bone signal and suppresses soft tissues.

A joint‐L_0_ wavelet regularization term enhances thin bony structures because it ensures that both echoes share the same set of nonzero wavelet coefficients [48]. It also leverages properties of sparsity‐promoting reconstruction to reduce total scan duration [35, 52]. Typical T_1_w UTE and CTE images and the resulting bone‐selective image are shown in Figure 3b.

### 2.3 DREAMER: Pulse Sequence and Image Reconstruction

**FIGURE 4:**
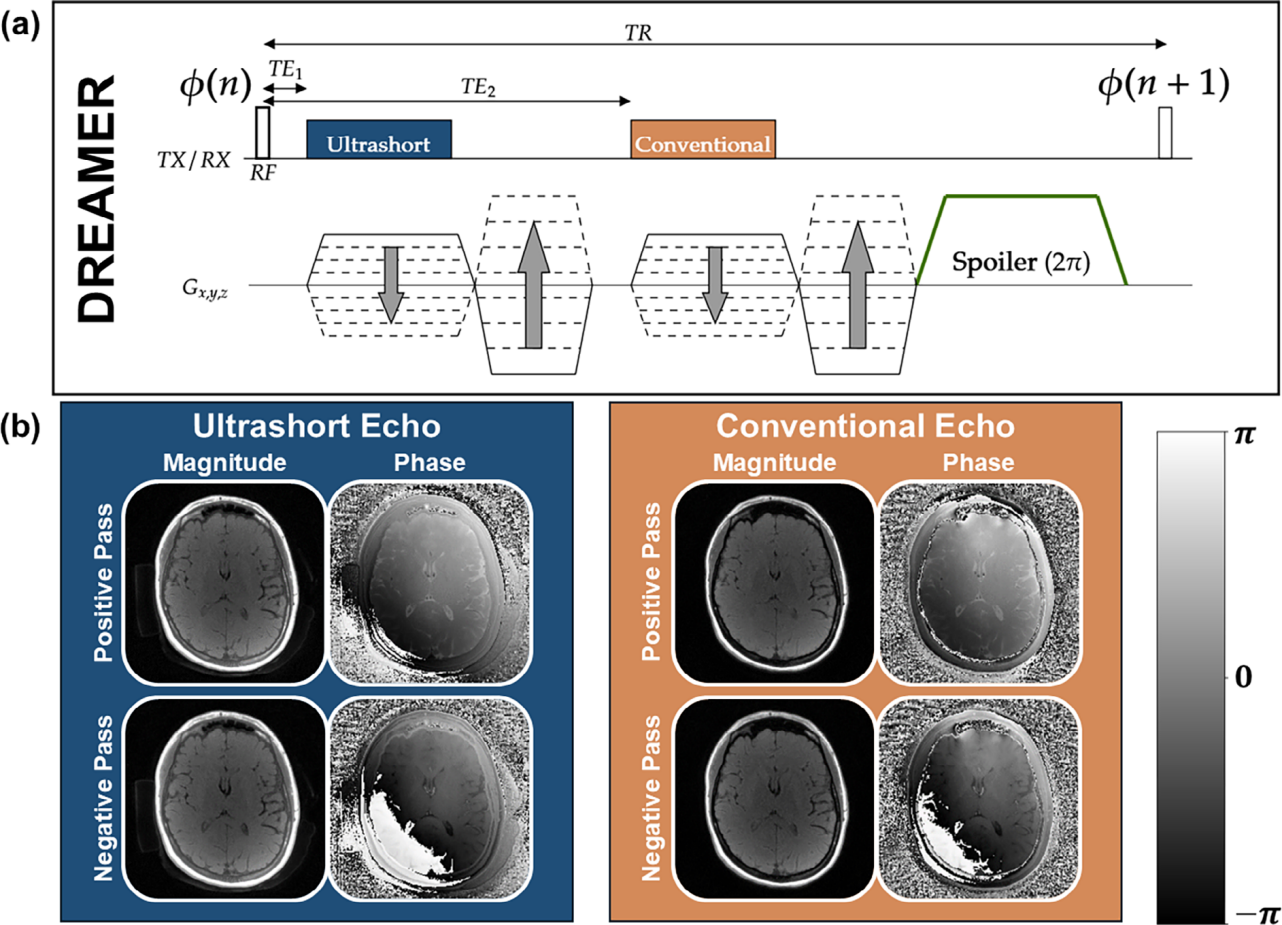
(a) Pulse sequence diagram of DREAMER (Dual Repetition and Echo Acquisition with Multi‐contrast Encoding and Reconstruction). DREAMER combines the image contrast mechanisms of the PSGRE and dual‐echo UTE parent sequences. Each RF pulse in DREAMER is followed by two echo acquisitions (one ultrashort, one conventional) to sensitize the sequence to short‐T_2_ (bone‐selective) signal. The phase schedule ϕ(n) uses a small increment to encode T_2_‐weighting along the signal phase for long‐T_2_ (soft tissue) species. Two repetitions (one positive and one negative RF phase increment) are acquired to correct for the background phase. (b) A single DREAMER scan produces four datasets (2 echoes × 2 passes), which are used to compute two multi‐contrast images (ultrashort and conventional echo). The vertical phase scale gives the values for the image phase in radians.

**FIGURE 5:**
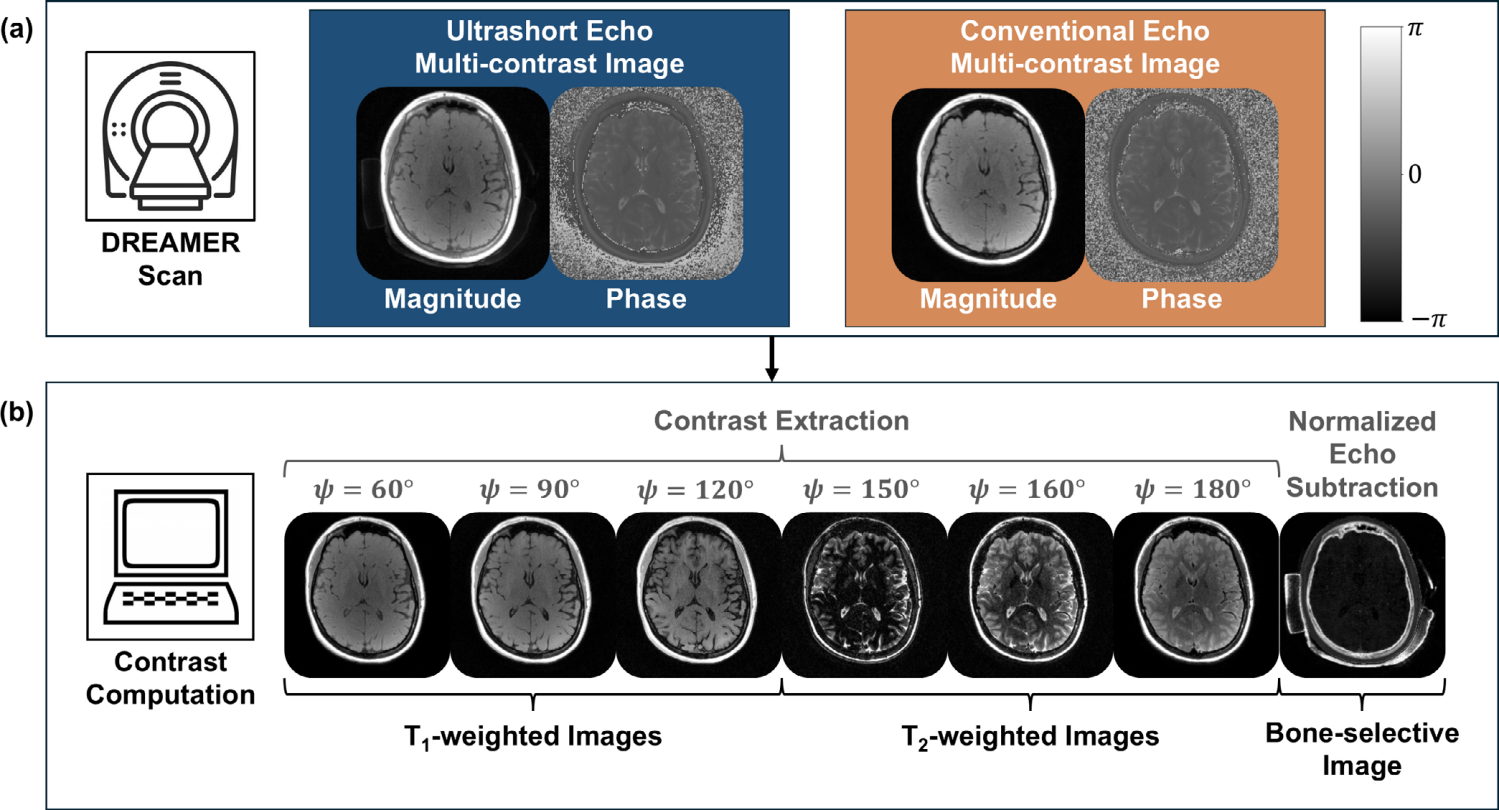
Image reconstruction of solid and soft tissue image contrasts from a single MRI scan with DREAMER. (a) DREAMER produces ultrashort and conventional echo multi‐contrast images with and without bone‐selective signal, respectively. The vertical phase scale gives the values for the image phase in radians. (b) From the two multi‐contrast images all image contrasts can be computed. Phase modulation ψ enables tunable soft tissue contrast extraction of T_1_w and T_2_w images. A normalized echo subtraction between the two multi‐contrast images yields a bone‐selective image in which short‐T_2_ species are enhanced.

DREAMER uses contrast mechanisms from parent PSGRE and dual‐echo UTE sequences to achieve simultaneous bone‐selective, T_1_w, and T_2_w imaging. The pulse sequence diagram for DREAMER is shown in Figure 4a. In each pass (positive and negative), a train of RF pulses is applied with a small RF phase increment to concurrently encode T_1_‐ and T_2_‐weighting along the steady‐state signal phase. Ultrashort and conventional echoes are acquired following each RF pulse to also sensitize the sequence to short‐T_2_ signal. A single DREAMER acquisition produces four phase‐corrupted images (2 echoes × 2 passes; Figure 4b). Analogous to a standard dual‐echo sequence, which produces two T_1_w images and one bone‐selective image, DREAMER produces two multi‐contrast images (Figure 5a) and one bone‐selective image.

Image reconstruction from DREAMER data follows a similar path to that of the parent sequences (Figure 5b). Four phase‐corrupted multi‐contrast images are reconstructed from the four acquired k‐space datasets:

(12) $$\begin{array}{c} \left(x_{k,l}\right)=\underset{\left(u_{k,l}\right)}{\text{argmin}}\;\sum_{k=\left\{\mathrm{UTE},\mathrm{CTE}\right\}}\;\sum_{l=\left\{+,-\right\}}{\left\|\mathrm{FS}u_{k,l}-y_{k,l}\right\|}_{2}^{2}+R\left(u_{k,l}\right) \end{array}$$

Indices k={UTE,CTE} and l={+,−} are used for compactness and to denote association with the echo (ultrashort or conventional) and steady‐state pass (positive or negative). Equation (12) reconstructs a pair of positive and negative images for each of the two acquired echo times by enforcing data consistency on each k‐space dataset. Like with dual‐echo UTE reconstruction, a joint‐L_0_ wavelet regularization term $R\left(u_{k,l}\right)$ is used across all four images to promote bone specificity in the normalized echo subtraction.

Following reconstruction of the four phase‐corrupted images, the background phases $\varphi_{\mathrm{UTE}}\left(r\right)$ and $\varphi_{\mathrm{CTE}}\left(r\right)$ are estimated for both the ultrashort and conventional echoes:

(13) $$\begin{array}{c} e^{i\varphi_{k}\left(r\right)}={\left(\frac{x_{k,+}}{x_{k,-}^{*}}\right)}^{\frac{1}{2}} \end{array}$$

The UTE and CTE multi‐contrast images $x_{\mathrm{UTE}}$ and $x_{\mathrm{CTE}}$ are then similarly calculated by averaging the phase‐corrected images from positive and negative passes:

(14) $$\begin{array}{c} x_{k}=\frac{1}{2}\left[x_{k,+}e^{-i\varphi_{k}\left(r\right)}+{\left(x_{k,-}e^{-i\varphi_{k}\left(r\right)}\right)}^{*}\right] \end{array}$$

Contrast extraction may be performed on either the UTE or CTE multi‐contrast image to yield T_1_w and T_2_w images,

(15) $$\begin{array}{c} x_{k,\psi }=\left|R\left(x_{k}e^{i\psi }\right)\right| \end{array}$$

and a bone‐selective image $x_{s}$ is computed from both the UTE and CTE multi‐contrast images by the direct normalized echo subtraction method [45] in Equation (9) or by the weighted least‐squares method [48] in Equation (10). The present study performs contrast extraction on the CTE multi‐contrast image and exclusively uses the weighted least‐squares method for computing the bone‐selective image.

### 2.4 Imaging Details

MRI was performed at 3 T (Prisma, Siemens, Erlangen, Germany). Subjects were scanned at the head using a 64‐channel head‐and‐neck coil. Two healthy female adult subjects (ages 24 and 29 years old) were recruited to receive head and knee MR imaging for this study. In addition, two pediatric patients (one 17 years old male and one 17 years old female) were recruited for additional research head MRI at the Children's Hospital of Philadelphia. CT images were acquired with an in‐plane voxel length of 0.42 × 0.42 mm^2^ and 0.75 mm slice thickness. All research imaging was performed after obtaining informed consent and in accordance with the University of Pennsylvania and Children's Hospital of Philadelphia's Institutional Review Board requirements.

DREAMER was implemented in SequenceTree [53] with a golden‐angle ordering [54] and a center‐out, ramp‐up radial trajectory. To assess the soft‐tissue contrast of DREAMER with clinical imaging methods, T_1_w MPRAGE and T_1_w and T_2_w fast spin‐echo (FSE) were compared with their counterparts in DREAMER, with voxel sizes matched to those of DREAMER. For the contrast comparisons, phase modulation values were selected to create a qualitative “best match” with the clinical standard. For bone‐selective contrast comparison, the patient's CT image was downsampled to an isotropic voxel size of 1 mm^3^ and registered with the DREAMER images. Sequence parameters are listed in Table 1. All reported scan times for DREAMER refer to the total duration of the sequence (i.e., both positive and negative passes).

**TABLE 1:** Sequence parameters used in this study.

Parameters	DREAMER (head)	T_1_w MPRAGE (head)	T_2_w FSE (head)
FOV	(280, 280, 280) mm	(256, 256, 256) mm	(256, 256, 75) mm
Flip angle	24°	9°	90°
RF phase increment	1.5°		
Refocusing flip angle			150°
TR	5.5 ms	1900 ms	5500 ms
TE	(80 μs, 2.38 ms)	2.28 ms	90 ms
TI		958 ms	
Pulse duration(s)	60 μs		
Readout ramp‐up time	160 μs		
Dwell time OR BW/pixel	4 μs	190 Hz/px	260 Hz/px
Matrix size	(280, 280, 280)	(256, 256, 256)	(256, 256, 75)
Voxel size	(1) mm^3^	(1) mm^3^	(1) mm^3^
GRAPPA factor		2, w/24 ref. lines	2, w/24 ref. lines
Echo train length			18
Concatenations			3
Scan duration	6.0 min for both passes (unless otherwise specified)	4.4 min	2.5 min

*Note*: All sequences for head imaging were acquired at the same voxel size (1 mm^3^ isotropic). Except for the voxel size and field‐of‐view, MPRAGE and T_2_w FSE sequence parameters were taken from a typical protocol used for pediatric head imaging at the Children's Hospital of Philadelphia. All head images were acquired using a 64‐channel head‐and‐neck coil.

Abbreviations: BW, bandwidth; D‐RF, dual radiofrequency; FOV, field‐of‐view; GRAPPA, GeneRalized Autocalibrating Partial Parallel Acquisition; px, pixel; ref., reference; TI, inversion time; w/, with.

Image reconstruction was implemented using the SigPy [55] library on a desktop workstation with an Intel Core i9‐10980XE, 256 GB RAM, and an NVIDIA Geforce RTX 2070 Super with 8 GB VRAM. The Flatiron Institute NUFFT was used for its computational efficiency and relatively low memory overhead [56]. Coil sensitivity maps were estimated by NLINV on the graphics processing unit [36, 55]. To accelerate convergence and reduce reconstruction time, k‐space preconditioning [57] was used with the primal‐dual hybrid gradient algorithm [58], which ran on the central processing unit. A U‐Net model [59] for skull segmentation, developed and trained in a prior study [48], was applied to DREAMER bone‐selective images to generate 3D skull renderings. These renderings were compared with 3D skull renderings generated from DURANDE [60] (dual‐RF, dual‐echo UTE) and CT acquisitions.

## 3 Results

**FIGURE 6:**
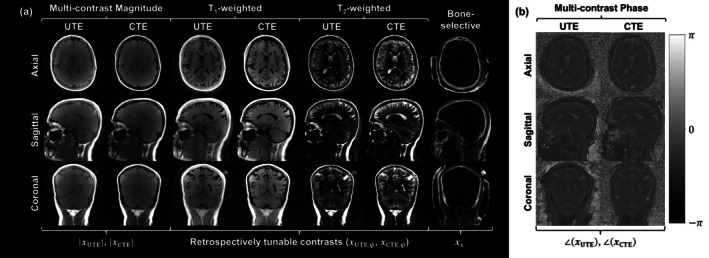
All representative contrasts of DREAMER for the three principal anatomical planes (24 years old healthy female). (a) A single DREAMER acquisition produces two 3D multi‐contrast images (ultrashort and conventional echoes). Although the multi‐contrast magnitude image is relatively featureless, T_1_w and T_2_w images ($\psi_{T1}=117{}^{\circ}$, $\psi_{T2}=156{}^{\circ}$), encoded along the signal phase, can be derived from either multi‐contrast image to substantially enhance the soft‐tissue contrast. Moreover, a bone‐selective image can be derived by performing a normalized echo subtraction between the ultrashort and conventional multi‐contrast images. Because the contrast mechanisms are concurrent, all image contrasts are self‐registered. (b) T_2_‐weighting is encoded in the phase of the background phase‐corrected multi‐contrast images. The vertical phase scale gives the values for the multi‐contrast image phase in radians.

Multi‐contrast magnitude and phase, T_1_w, T_2_w, and bone‐selective images are all acquired in a single DREAMER scan, demonstrated in a head scan of a healthy adult in Figure 6. For each DREAMER scan, two multi‐contrast images (UTE and CTE) form the basis for simultaneous T_1_w, T_2_w, and bone‐selective imaging. The image phase is an essential feature of the complex‐valued multi‐contrast images because it forms the basis for additional encoding of T_2_‐weighting (Figure 6b). Because both multi‐contrast images encode various contrast weightings in their phase, T_1_w and T_2_w images can be extracted from either one. A short‐T_2_ enhancing image highlighting bone is derived from a normalized echo subtraction between the ultrashort and conventional multi‐contrast images.

**FIGURE 7:**
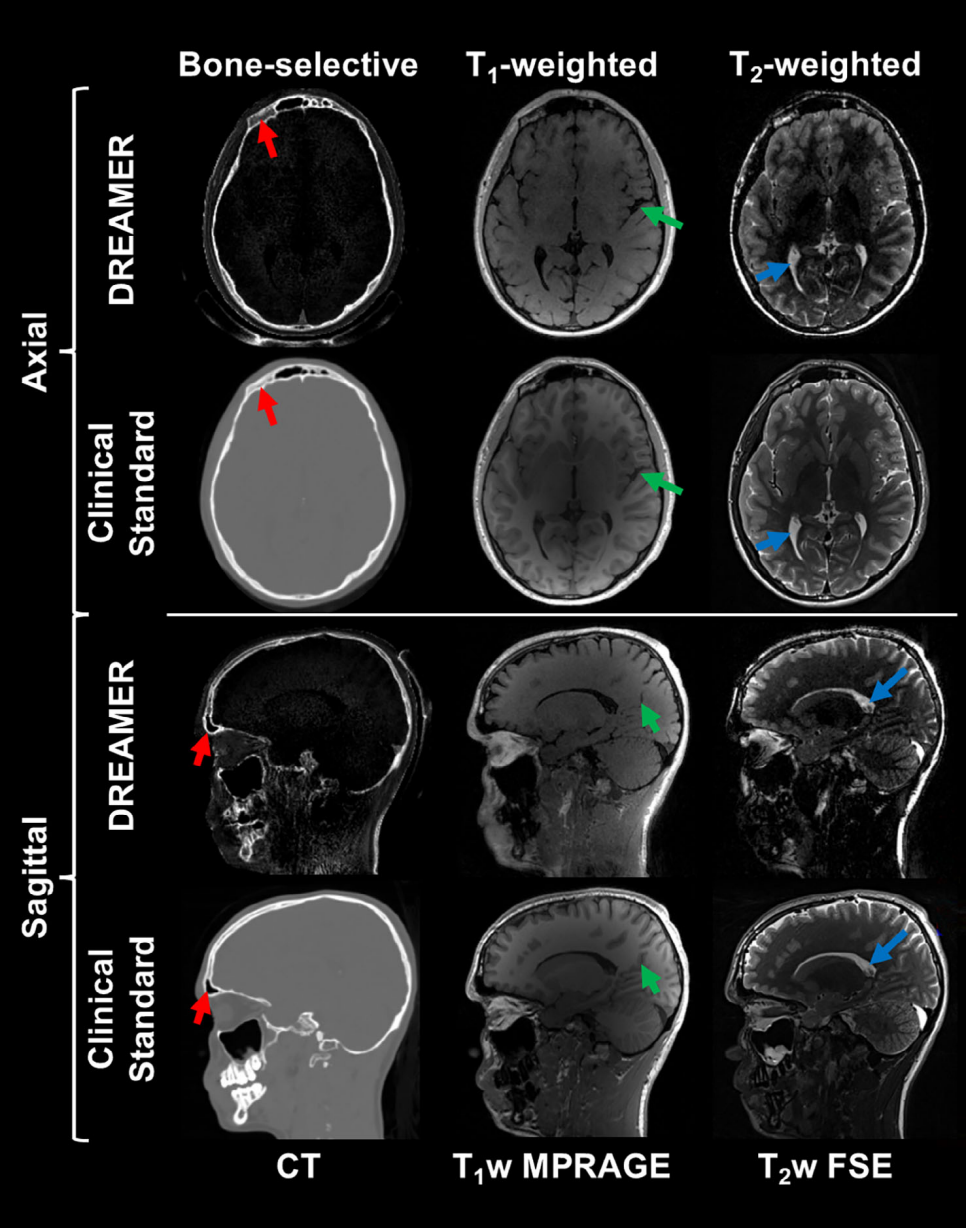
Comparison of DREAMER image contrasts with their corresponding clinical standards for a pediatric patient (17 years old male; 6 min total scan time for both positive and negative passes). Bone‐selective images are compared with CT, T_1_w DREAMER images with T_1_w MPRAGE, and T_2_w DREAMER images with T_2_w multi‐slice FSE ($\psi_{T1}=114{}^{\circ}$, $\psi_{T2}=159{}^{\circ}$). The same anatomical features are present in DREAMER images and their clinically standard counterparts (e.g., skull, brain sulci and gyri, and cerebrospinal fluid as red, green, and blue arrows). In a conventional head MRI protocol, T_1_w MPRAGE and T_2_w FSE scans are separately acquired, prolonging the total examination time. The additional acquisition of a CT image requires a separate examination, which increases patient wait time and complicates the clinical workflow.

Three contrasts commonly used for craniofacial imaging are included in a single DREAMER acquisition. A pediatric patient who had sustained trauma to the face 3 years ago was imaged with DREAMER, T_1_w MPRAGE, T_2_w FSE, and CT (Figure 7). The same anatomical features of the skull and brain are visible in both DREAMER images and the corresponding clinical standards. T_1_w DREAMER achieves less white‐gray matter contrast than T_1_w MPRAGE because the latter uses an inversion‐recovery technique to enhance T_1_‐weighting. Table S1 compares SNR and CNR measurements of T_1_w and T_2_w images.

The adjustable contrast feature of DREAMER images may enhance user sensitivity to differences in anatomy and tissue composition. Video S1 demonstrates post‐scan contrast adjustment. Various soft tissue structures are brought in and out of the viewer's focus as the phase modulation value is adjusted continuously from 0° to 180°.

**FIGURE 8:**
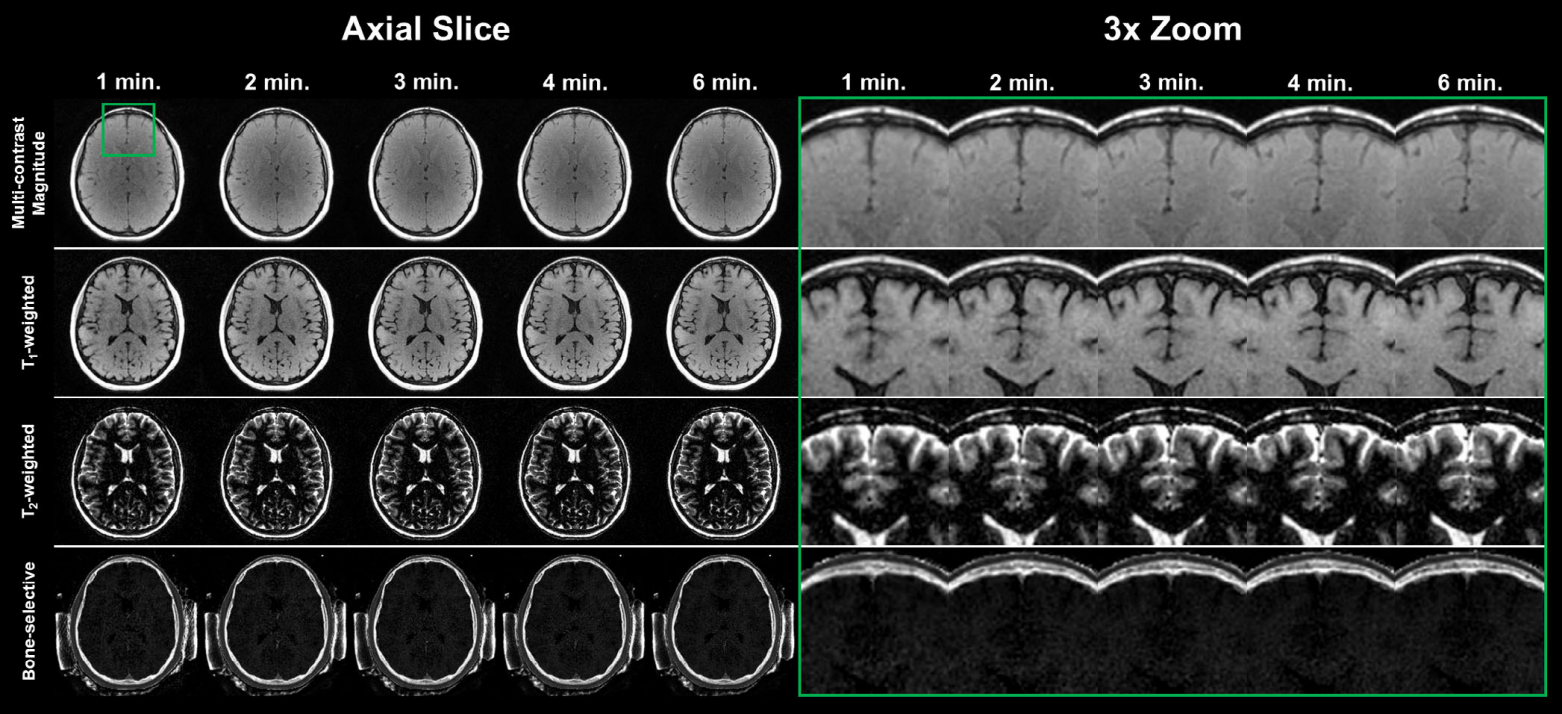
DREAMER image quality is robust to substantial reductions in scan duration. An axial slice of the brain and skull (29 years old healthy female) is shown for the four fundamental DREAMER image contrasts (multi‐contrast magnitude, T_1_w, T_2_w, and bone‐selective; $\psi_{T1}=114{}^{\circ}$, $\psi_{T2}=153{}^{\circ}$). Separate scans were performed with durations of 1, 2, 3, 4, and 6 min (i.e., total scan time for both positive and negative passes). Differences in image quality between the scans of varying duration can mostly be appreciated upon close inspection of a small region of interest (green box). As the scan duration decreases, the images undergo increased blurring and image details are obscured. However, this loss in image quality is minimal with respect to the 6‐min benchmark, even for scan durations of 2–3 min. Even for the shortest scan time (1 min), the inner and outer tables of the skull are delineated.

The solid‐ and soft‐tissue contrast mechanisms of DREAMER can be directly incorporated into a model‐based reconstruction. Model‐based reconstruction leverages improvements in receiver coil hardware and advances in imaging theory to achieve significant reductions in scan time [34, 36]. The four fundamental DREAMER contrasts, derived from the two multi‐contrast images, can be reconstructed from scans of varying duration (Figure 8). Image quality is reasonably maintained even for short scans of 2–3 min. However, as the scan duration decreases, blurring ensues and the apparent resolution decreases, becoming noticeable at very high acceleration factors (e.g., at 1–2 min). A short scan yielding several isotropic and co‐registered solid and soft tissue images over a large three‐dimensional field‐of‐view may increase the value proposition of MRI over CT.

**FIGURE 9:**
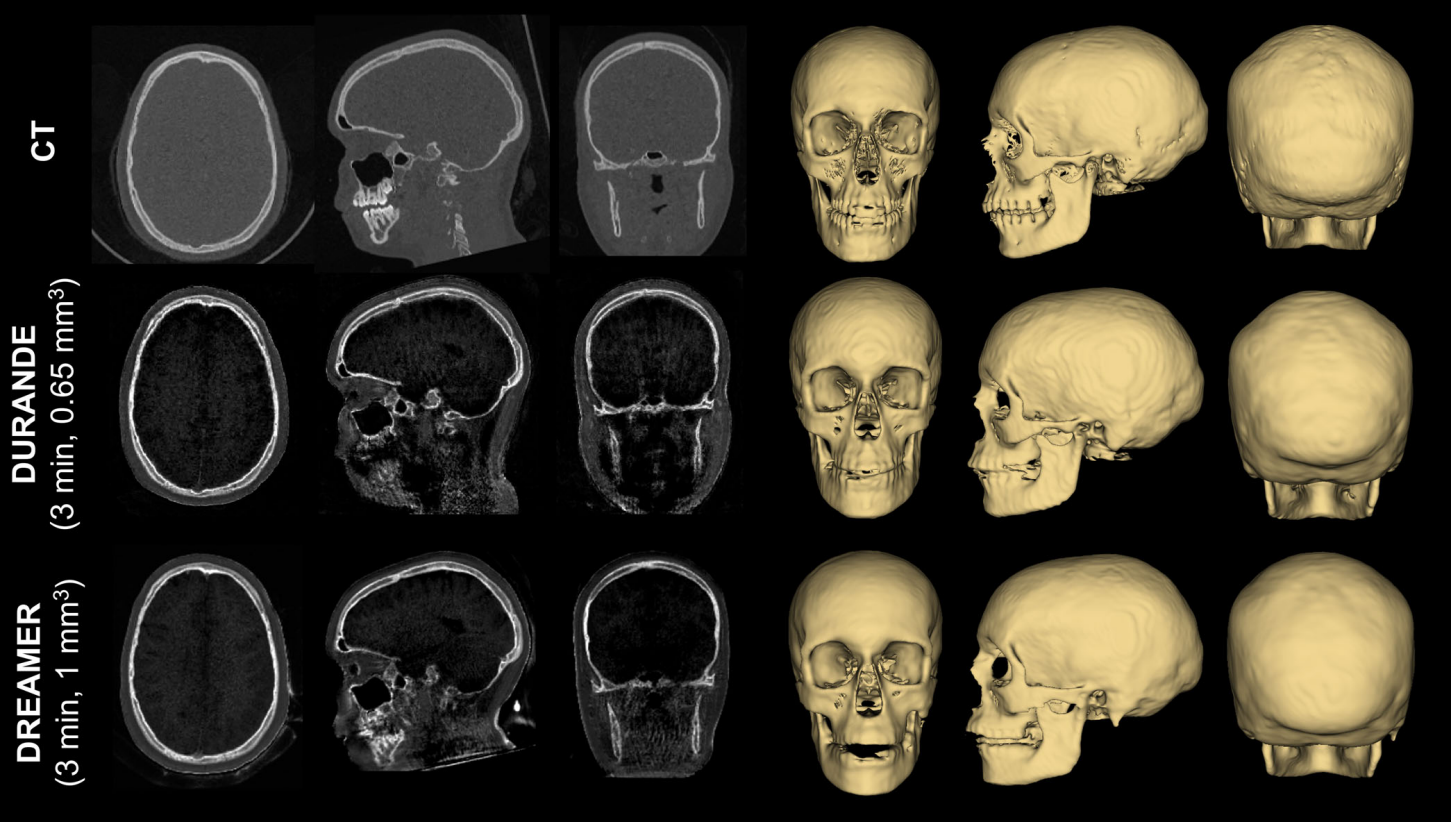
DURANDE (dual‐RF, dual‐echo UTE) and DREAMER bone‐selective images validated with CT for a 17‐year‐old female pediatric patient with fibrous dysplasia and no history of prior cranial vault surgery. DURANDE and DREAMER share the same contrast mechanism for bone‐selective imaging, and hence, full 3D skull renderings from the MR bone‐selective images can be generated from the same U‐Net model previously trained only on DURANDE images.

Because the bone‐selective contrast mechanism of DREAMER is identical to that of a dual‐echo UTE sequence, previously developed image processing tools in the authors' laboratory for bone‐selective imaging [48] may be applied to DREAMER images without modification. Figure 9 compares bone‐selective images and renderings, derived from CT, DURANDE [60] (i.e., a dual‐echo UTE sequence), and DREAMER for a pediatric patient with a clinical history of fibrous dysplasia. The U‐Net model [59], developed and used in a previous study [48] to segment DURANDE bone images, was applied to DREAMER bone images without additional training (i.e., U‐Net training data consisted of only DURANDE images, as in the previous study, and did not include any DREAMER data). A qualitative comparison of the anatomy depicted in the bone‐selective images and 3D skull renderings suggests that sequence differences between DURANDE and DREAMER do not substantially and negatively impact the bone‐selective contrast mechanism nor the segmentation model performance.

Reconstruction times increased with the amount of raw k‐space data collected (i.e., the duration of the scan). Precise timing benchmarks are as follows for 3‐ and 6‐min DREAMER scans, respectively: coil sensitivity map estimation, 1.8 and 3.4 min; image reconstruction of the four phase‐corrupted multi‐contrast images with joint wavelet regularization, 104.7 and 134.4 min; reconstruction of the bone‐selective image, 0.3 and 0.3 min. The time calculating the background phase and extracting soft‐tissue contrasts was negligible because these are element‐wise array operations.

## 4 Discussion

This study proposed a new MRI pulse sequence, DREAMER, to simultaneously acquire solid and multiple‐contrast soft tissue images of the skull and brain. DREAMER images are 3D, isotropic in resolution, and self‐registered. DREAMER multi‐contrast images enable retrospective and user‐customizable weightings of T_1_ and T_2_, giving clinicians a potential tool to more sensitively detect and diagnose pathology. In the brain and skull, DREAMER achieves similar soft tissue contrast to standard clinical MRI techniques (T_1_w MPRAGE and T_2_w FSE) and acquires solid‐tissue structural information comparable to CT. Advanced reconstruction techniques [34, 35, 36, 48] are used with DREAMER to reduce scan time without substantially compromising image quality. In its present form, DREAMER lays the foundation for a radiation‐free imaging technique that consolidates many of the beneficial characteristics of conventional craniofacial MRI and CT.

In addition to integrating the PSGRE and dual‐echo UTE contrast mechanisms to create DREAMER, the present work builds upon previously established theory for the PSGRE and dual‐echo UTE sequences. Section 2.1 (PSGRE) states the forward model for simultaneous T_1_w and T_2_w encoding. New contributions relative to the works of Tamada et al. [43] and Wang et al. [41] include explicitly modeling and solving for the background phase and multi‐contrast image in the presence of coil sensitivities and arbitrary Fourier encoding (Equations (4), (5a), and (5b)). This work extends the dual soft‐tissue contrast mechanism of PSGRE to non‐Cartesian and arbitrarily undersampled trajectories. Unlike the work by Tamada et al. [43], the present work explicitly computes the background phase and multi‐contrast image, the latter a spatially discretized representation of the superposition of FID, spin‐echo, and stimulated‐echo magnetization components in each voxel resulting from the PSGRE sequence. This work also shows that all soft‐tissue image contrasts are extracted for phase modulation values ψ in the interval $\left[0{}^{\circ},180{}^{\circ}\right)$ (Equation (3)).

Section 2.2 (Dual‐echo UTE) provides a new analysis of the signal behavior of long‐T_2_ (T_2_ ≫ TR) and short‐T_2_ (T_2_ ≪ TR) signals in the context of an RF pulse train with arbitrary phase increment. In Figure 2a, it is shown that the steady‐state signal magnitude of long‐T_2_ signals varies with a changing RF phase increment, because a zero or small θ retains residual transverse magnetization and permits a non‐negligible contribution of T_2_‐weighting to the overall signal. On the other hand, the transverse magnetization of short‐T_2_ signals is completely or near‐completely spoiled before the end of the TR block, and hence, no residual transverse magnetization is refocused by the subsequent RF pulse. Therefore, short‐T_2_ magnetization components cannot accrue phase over the course of multiple TR blocks, and the steady‐state signal magnitude of short‐T_2_ signals is indifferent to the choice of RF phase increment. Hence, bone‐selective UTE imaging is unaffected by a change in the RF phase schedule, offering complete flexibility in the choice of θ used in the quadratic RF phase increment (Equation (1)) for dual‐echo UTE sequences. A small, nonzero θ can be utilized in the PSGRE portion without compromising the bone‐selective imaging capabilities. The DREAMER technique is the result of combining this insight on short‐T_2_ signals with the new reconstruction techniques developed for PSGRE sequences (Section 2.3).

DREAMER's bone‐selective contrast mechanism is identical to that of a standard dual‐echo UTE. Bone‐selective imaging by normalized echo subtraction was previously evaluated in pediatric patients assessing surgically relevant craniometric distances and segmentation accuracy when comparing MR images and CT images [51]. Moreover, because DREAMER produces UTE and CTE T_1_w contrasts and a bone‐selective contrast (among many others), DREAMER images may be segmented with the U‐Net algorithm described by Vu et al. [48], which was trained solely on standard dual‐echo UTE images and did not see DREAMER data during training. A future study will further investigate DREAMER's feasibility in pediatric patients indicated for head CT and MRI, including evaluation of image quality by a radiologist and direct comparison with CT and previously established craniofacial bone MRI methods (e.g., ZTE and black‐bone MRI).

In comparison to T_1_w MPRAGE, DREAMER T_1_w images depicted lower white‐gray matter contrast because the former employs an inversion pulse to enhance T_1_‐weighting. However, inversion pulses reduce the scan efficiency of a sequence and prolong scan time. DREAMER, instead, operates in a steady state, allowing for repeated readouts without needing to wait for the recovery of magnetization, a useful property for rapid pediatric craniofacial imaging in which soft‐tissue contrasts are sometimes used to identify gross abnormalities secondary to skull deformities (e.g., hydrocephalus, Chiari malformations). Moreover, DREAMER acquires solid and multiple soft‐tissue contrasts all at once, while dedicated single‐contrast sequences need to be acquired separately.

While this work focused on implementing DREAMER for fast craniofacial imaging of pediatric patients, a fat suppression mechanism is essential for extending DREAMER to musculoskeletal applications. For solid‐tissue imaging, fat suppression requires no modification to the sequence or reconstruction—a normalized echo subtraction [45] removes proton density weighting to overcome some of the signal limitations inherent to cortical bone water imaging (∼20% proton density by volume [47]). Normalization also effectively suppresses high proton density fat signal in bone‐selective images [37]. To suppress fat in soft‐tissue images, a multipoint Dixon strategy, operating in the PSGRE steady‐state, may be employed to separate fat and water signal [43, 61, 62, 63]. This would likely entail modifying DREAMER to contain multiple echo times to sample the in‐ and out‐of‐phase timepoints of lipid and water protons at 3 T. However, this modification would also increase the TR and overall scan time.

Several limitations should be addressed in future work. DREAMER uses two passes to estimate and remove the background phase. A second pass creates a scan inefficiency and renders the sequence susceptible to patient motion between the two passes, which may result in an incorrect background phase estimation and an inability to correctly perform contrast extraction. Even if there is negligible patient motion, the large spoiler gradient used at the end of each TR block may generate eddy currents and cause the background phase to vary temporally. In both cases, the scan acceleration techniques used in this study mitigate the inefficiencies and weaknesses of a two‐pass strategy. A reduced scan duration decreases the likelihood of patient motion, and if the background phase varies temporally, a shorter scan time supports the assumption of a temporally static background phase. While the effects of subject motion and eddy currents in this work were found to be negligible and the multi‐contrast imaging capabilities of DREAMER were condensed to a single 3‐ to 6‐min total scan duration, future work will investigate methods to remove or further accelerate the second pass in DREAMER, such as by optimizing the encoding trajectory with spiral readouts [64, 65] and incorporating deep learning priors for image reconstruction [32]. Additionally, future studies should investigate the effect of eddy currents generated by the large spoiler gradients and the possibility of retrospective motion correction via the golden‐angle sampling strategy currently employed by DREAMER [66].

DREAMER image reconstruction in this study is computationally expensive and will require optimization for prospective studies involving patients. The main computational task in the reconstruction algorithm was the repeated application of forward and adjoint non‐uniform FFT (NUFFT) operators. Potential optimizations include GPU implementations of the NUFFT [67], the Toeplitz trick for non‐Cartesian imaging [68, 69], and coil compression [70] or coil sketching [71]. Nevertheless, the present work demonstrates feasibility for a rapid, single‐scan sequence capable of multi‐contrast solid‐ and soft‐tissue imaging.

Mineralized dental tissues appear with noticeably lower quality than cranial bone. The ultrashort TE used in DREAMER for this study (80 μs) may be too long for imaging dentin and enamel, which are known to have very short T_2_* values [72, 73, 74]. Further shortening of the ultrashort TE and optimization of spatial encoding gradients is necessary to improve the visual quality of dental tissues.

Finally, GRE or GRE‐derived sequences with unbalanced gradient moments, like DREAMER, are sensitive to flow artifacts. In the head, cerebrospinal fluid flows between the ventricles and the spinal cord. For sufficiently high flow velocity, signal voids may appear where fluid is expected due to deviations in RF spoiling from the prescribed RF phase schedule [75]. Future work will explore methods to reduce these undesirable artifacts.

In summary, DREAMER may be a viable alternative to clinical CT and conventional clinical MRI for pediatric craniofacial imaging. DREAMER combines solid‐ and multiple‐contrast soft‐tissue imaging capabilities into a single pulse sequence. For imaging centers with high patient throughput, the development of DREAMER may serve as a foundational step in consolidating MRI and CT examinations and streamlining radiological workflows. For patients and clinicians, DREAMER may offer a one‐stop shop for structural solid and soft‐tissue craniofacial imaging.

## Supporting information


**Table S1:** SNR and CNR measurements of T1w MPRAGE, T2w FSE, and T1w and T2w DREAMER soft‐tissue images. SNR of T1w images was measured as white matter (WM) signal divided by the standard deviation of noise. SNR of T2w images was measured as the cerebrospinal (CSF) signal divided by the standard deviation of noise. CNR was measured between WM and gray matter (GM) and between CSF and WM (i.e., signal difference between tissues divided by the standard deviation of noise). Measurements of SNR and CNR were made by manually selecting regions of interest across multiple axial slices for the two pediatric patients in this study. The reported values are average values of the two patients' measurements.

**Video S1:** Extracted soft‐tissue image contrasts (top row) and bone‐selective images (bottom row) for axial (left column), coronal (middle column), and sagittal (right column) slices of a 3D DREAMER acquisition (24 year old healthy female). For the soft‐tissue image contrasts, the video advances the phase modulation parameter *ψ* starting from *ψ* = 0° to *ψ* = 180° at a rate of 6° per second.
