# Supplementary material for: DREAMER: Rapid and Simultaneous Multiple Contrast Magnetic Resonance Imaging of Solid and Soft Tissue
Source: Magn Reson Med. 2025 Oct 29;95(3):1513–27. doi: 10.1002/mrm.70140 (PMC12746388; doi:10.1002/mrm.70140)
Supplement: Supplementary file 1 — Table S1: SNR and CNR measurements of T1w MPRAGE, T2w FSE, and T1w and T2w DREAMER soft‐tissue images. SNR of T1w images was measured as white matter (WM) signal divided by the standard deviation of noise. SNR of T2w images was measured as the cerebrospinal (CSF) signal divided by the standard deviation of noise. CNR was measured between WM and gray matter (GM) and between CSF and WM (i.e., signal difference between tissues divided by the standard deviation of noise). Measurements of SNR and CNR were made by manually selecting regions of interest across multiple axial slices for the two pediatric patients in this study. The reported values are average values of the two patients' measurements. [file MRM-95-1513-s002.docx]

**Table S1:** SNR and CNR measurements of T_1_w MPRAGE, T_2_w FSE, and T_1_w and T_2_w DREAMER soft-tissue images. SNR of T_1_w images was measured as white matter (WM) signal divided by the standard deviation of noise. SNR of T_2_w images was measured as the cerebrospinal (CSF) signal divided by the standard deviation of noise. CNR was measured between WM and gray matter (GM) and between CSF and WM (i.e. signal difference between tissues divided by the standard deviation of noise). Measurements of SNR and CNR were made by manually selecting regions of interest across multiple axial slices for the two pediatric patients in this study. The reported values are average values of the two patients’ measurements.

|  | T_1_w MPRAGE | T_2_w FSE | T_1_w DREAMER | T_2_w DREAMER |
| --- | --- | --- | --- | --- |
| SNR (WM/noise) | 93.7 |  | 25.8 |  |
| SNR (CSF/noise) |  | 101.4 |  | 35.2 |
| CNR (WM & GM) | 38.1 | 21.5 | 2.9 | 14.0 |
| CNR (CSF & WM) |  | 80.1 |  | 29.7 |

**Video S1**: Extracted soft-tissue image contrasts (top row) and bone-selective images (bottom row) for axial (left column), coronal (middle column), and sagittal (right column) slices of a 3D DREAMER acquisition (24 y.o. healthy female). For the soft-tissue image contrasts, the video advances the phase modulation parameter $\psi$ starting from $\psi=0^{\circ}$ to $\psi=180^{\circ}$ at a rate of $6^{\circ}$ per second.
